# Redox Cycling Realized in Paper-Based Biochemical Sensor for Selective Detection of Reversible Redox Molecules Without Micro/Nano Fabrication Process

**DOI:** 10.3390/s18030730

**Published:** 2018-02-28

**Authors:** So Yamamoto, Shigeyasu Uno

**Affiliations:** Department of Electrical and Electronic Engineering, Ritsumeikan University, Kusatsu, Shiga 525-8577, Japan; re0065xk@ed.ritsumei.ac.jp

**Keywords:** redox cycling, electrochemical sensor, chromatography paper, paper-based sensor, ascorbic acid

## Abstract

This paper describes a paper-based biochemical sensor that realizes redox cycling with close interelectrode distance. Two electrodes, the generator and collector electrodes, can detect steady-state oxidation and reduction currents when suitable potential is held at each electrode. The sensor has two gold plates on both sides of a piece of chromatography paper and defines the interelectrode distance by the thickness of the paper (180 μm) without any micro-fabrication processes. Our proposed sensor geometry has successfully exhibited signatures of redox cycling. As a result, the concentration of ferrocyanide as reversible redox molecules was successfully quantified under the interference by ascorbic acid as a strong irreversible reducing agent. This was possible because the ascorbic acids are completely consumed by the irreversible reaction, while maintaining redox cycling of reversible ferrocyanide. This suggests that a sensor based on the redox cycling method will be suitable for detecting target molecules at low concentration.

## 1. Introduction

The number of patients with chronic disease has been increasing due to unhealthy lifestyle habits [1,2]. Thus, a daily health check is recommended for prevention and control. Health condition can be checked by measuring various chemical species as biomarkers contained in the body fluid [3,4,5]. Such biomarkers can be detected quantitatively by electrochemical sensors [6]. Since some target molecules exist at low concentrations, highly sensitive measurements are required [7,8,9]. In addition, selectivity is also indispensable. Some chemical species interfere with the chemical reaction of the target molecules and make electrochemical measurement difficult [8,9,10,11,12]. For example, oxidation of ascorbic acid (AA) may occur simultaneously with oxidation of dopamine because the redox potentials of these molecules are close to each other (+0.2 V vs. Ag/AgCl) [8,11].

Highly sensitive and selective measurement can be realized by redox cycling [8,13,14,15,16,17,18]. This phenomenon is utilized for chemically reversible redox species (reversible species) in cases where the distance between two working electrodes (generator-collector electrode system) is of μm-order. In contrast, it does not occur in chemically irreversible redox species (irreversible species) such as AA, because the oxidized species converts rapidly to a chemically inactive species. Thus, the measurement of reversible species under redox cycling is possible regardless of the coexisting irreversible species. In fact, selective detection of reversible species for 100 times AA has been reported [19]. Furthermore, highly sensitive measurement is realizable, since redox cycling allows current amplification [8,14,15,20]. The major parameters of such a redox cycling system include the distance between the electrodes and the collection efficiency [21]. In redox cycling studies, interdigitated array electrodes are frequently used, which consist of two or more closely spaced co-planar linear electrodes [8,13,14,17,22]. In such interdigitated electrodes, molecules generated by one electrode are efficiently collected by the other electrode. There are other reports based on nanopore structures [23] or dual-plate micro-trench structures [24,25,26,27], realizing good sensitivity by electrodes as close as 10 μm. However, these electrodes require expensive and complicated micro-fabrication processes.

With the aim of avoiding such micro-fabrication processes, there have been reports of building redox cycling systems using micro-scale beads as spacers [28,29]. These realized sub-10 μm gaps between two electrodes without any micro-fabrication processes. Catechol-modified chitosan systems have also been reported as a bio-fabrication method for realization of redox cycling systems [30].

Meanwhile, sensors using a piece of paper have gained attention due to their usability, low cost, and disposability [31,32,33,34]. We refer to such a sensor as a Paper-based Biochemical Sensor (PBBS), and we are conducting research aiming for the simplification of the manufacturing process and the reduction of cost. Previously, we proposed a novel electrode structure for redox cycling, where the interelectrode distance was as small as 180 μm by separating two electrodes with the paper thickness [35]. The most notable feature of this sensor is that the paper acts not only as a supporter of the sample solution and the associated chemical reaction, but also as a spacer between two electrodes. Our sensor showed redox cycling even without any micro-fabrication processes, and we concentrated on the enhancement of the electrochemical current by redox cycling [35]. In order to prove the usefulness of our PBBS for redox cycling, however, we need to demonstrate selective measurement of reversible species in the presence of interference by irreversible species.

In this paper, we demonstrate quantitative detection of ferrocyanide as reversible species in the presence of ascorbic acid (AA) as irreversible species under redox cycling condition using the previously proposed electrode structure. We use the thickness of a piece of chromatography paper to define the micro-scale gap between two electrodes needed for redox cycling. Although our proposed sensor may ultimately be used for ultra-sensitive detection of biomolecules at very low concentrations, such as dopamine, we focus our efforts here on demonstration of selective detection of reversible species under interference by irreversible species. Therefore, we used ferrocyanide and ascorbic acids as a model case to reveal the operation principles of our PBBS. We first show by cyclic voltammetry measurements of ferrocyanide solution that PBBS with two electrodes separated by the thickness of the chromatography paper (ChrPr) displays redox cycling. We then show by chronoamperometry measurements of ferrocyanide/AA mixture solution that redox cycling enables quantification of ferrocyanide concentration even under the interference by AA.

The rest of the paper is organized as follows: Section 2 shows the theory for this experiment, Section 3 presents the experimental method, the results and the discussion are shown in Section 4, and finally, Section 5 gives the conclusion of this paper.

## 2. Theory

**Figure 1:**
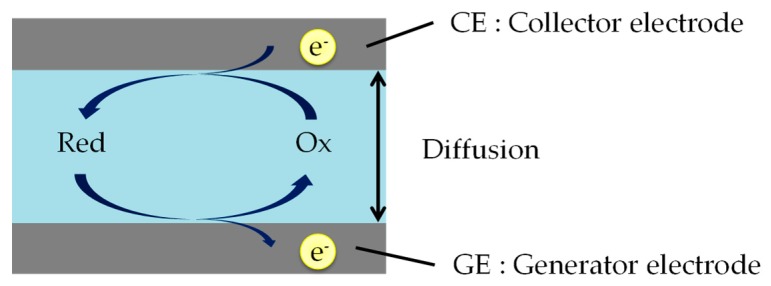
The schematic of the redox cycling method. Red and Ox are reduced and oxidized species. Reduced species are oxidized on GE surface region, and soon afterwards are re-reduced on CE surface region. In this case, the reaction at CE (e.g., Ox + e^−^ → Red) is just the reverse of that at GE (e.g., Red → Ox + e^−^).

Redox cycling needs two working electrodes, a generator electrode (GE) and a collector electrode (CE). As shown in Figure 1, the two electrodes measure oxidation and reduction currents when those are held at positive (oxidative) and negative (reductive) potentials with respect to the redox potential.

In this study, redox cycling is evaluated by two types of measurement techniques, cyclic voltammetry (CV) and chronoamperometry (CA). Each measurement observes electrochemical current from chemical reaction that occurs on the surface of the working electrode when a potential is applied to these electrodes.

**Figure 2:**
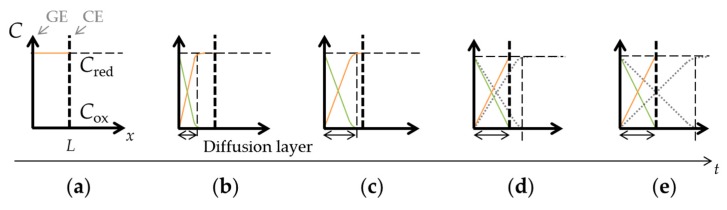
Concentration-distance (*C*−*x*) profiles under redox cycling: (**a**) a state in which the Red is filled; (**b**) the concentration gradient that occurred because of the oxidation reaction at the GE; (**c**) the growing diffusion layer with time; (**d**,**e**) the diffusion layer limited by interelectrode distance.

In the CV measurement, a cyclically varying potential is applied to the working electrode with respect to a reference electrode, and the electrochemical current at the working electrode is measured. The CV current is governed by both the electron-transfer and mass-transfer (diffusion) processes. On the other hand, in the CA measurement, the potential is kept constant during the electrochemical current measurement. In this case, the current is mainly governed by the diffusion process after a certain amount of time, and it is given by the following Cottrell’s equation [36]:

(1) $$I=nFAC_{\mathrm{bulk}}\sqrt{\frac{D}{\pi t}},$$

where *I* is the current through the working electrode, *n* is number of electrons transferred per molecule, *F* is the Faraday constant, *A* is the surface area of the working electrode, *C*_bulk_ is the concentration of target molecules in bulk solution, *D* is the diffusion coefficient, and *t* is the time after the constant voltage application. The current is proportional to the concentration gradient at the electrode surface due to diffusion of the target molecules, and is inversely proportional to the square root of time. The diffusion layer, where a concentration gradient occurs as illustrated in Figure 2b,c, continues to grow with time. The diffusion layer thickness, *d*, is given by the following equation [36]:

(2) $$d=\sqrt{\pi Dt}.$$

In the case of close interelectrode distance of μm-order, the target molecules are oxidized at the GE and these oxidized molecules diffuse to the CE. Here, the oxidized molecules will be reduced back to the original form by the CE and diffuse back to the GE. Since the redox reaction occurs continuously at two electrodes, the concentration profiles of the reduced and oxidized target molecules will eventually be linear and unchanged with time in steady-state, as shown in Figure 2d,e. The thickness of the diffusion layer will be fixed by the interelectrode distance, *L*. Thus, concentration gradient is defined solely by the interelectrode distance, and the resulting steady-state current is expressed as follows:

(3) $$I=nFAC_{\mathrm{bulk}}\frac{D}{L}.$$

Equation (3) shows that the current is proportional to the inverse of *L* and is independent of time. Note that redox cycling occurs not on irreversible species but on reversible species, because simultaneous conversion between reduced and oxidized forms at the two electrodes is necessary for the steady-state cycling.

In our experiment, we used potassium ferrocyanide as the reversible species, and AA as the irreversible species. Ferrocyanide will be reversibly oxidized/reduced by GE/CE, and hence it will exhibit the redox cycling. This can be observed as steady-state currents at both the GE and the CE, and such currents will be the same in magnitude. The AA will also be oxidized by the GE surface, and the oxidized AA, namely dehydroascorbic acid (DHA), will be produced. This reaction will be observed as an electrochemical current at GE. However, as the DHAs will not be re-reduced back to AA, the current associated with AA will not be observed at CE. The conversion from AA to DHA will therefore continue until all AA molecules are consumed. Thus, occurrence of the reduction current at CE will be the sign of redox cycling of the ferrocyanide even in the presence of AA, realizing selective detection of the ferrocyanide.

Along with the above-mentioned reactions associated with CE and GE, direct interaction between ferrocyanide and AA may also occur. As soon as the ferricyanide (oxidized form of ferrocyanide) is produced by the oxidation reaction at GE, it can be reduced back to ferrocyanide using AA as a reducing agent [37].

ferrocyanide ⇌ ferricyanide + e^−^, (4)

ascorbic acid → dehydroascorbic acid + 2H^+^ + 2e^−^. (5)

This reaction will last until all the AA is completely consumed. This reaction will add complication to the interpretation of the measured oxidation/reduction currents observed at GE/CE by affecting the current magnitudes or the time needed before the steady-state, as demonstrated in Appendix A. However, this complication will not affect the steady-state current by redox cycling, because AA will be fully depleted by then. Therefore, selective detection of ferrocyanide concentration by observing reduction current at CE will be valid.

## 3. Experimental Method

**Figure 3:**
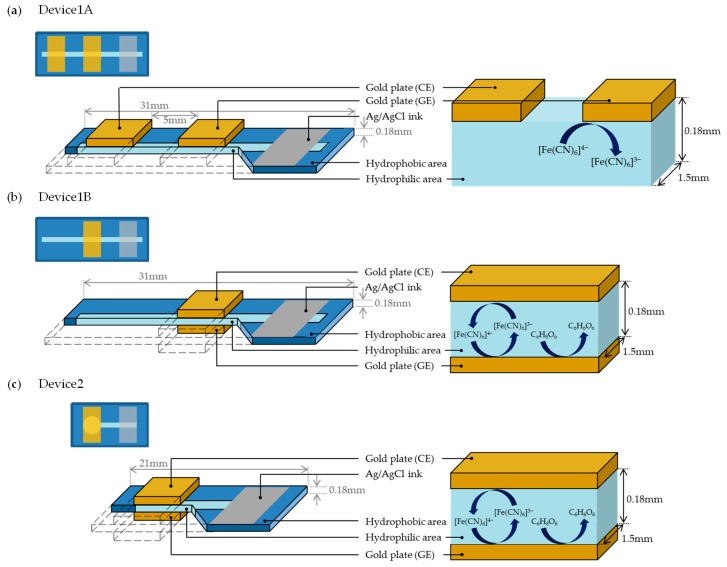
The schematic diagram and cross-sectional views: (**a**) Device1A, (**b**) Device1B, and (**c**) Device2.

We prepared three types of PBBS, which are referred to as Device1A, Device1B, and Device2, as shown in Figure 3. Each of these sensors consists of a piece of chromatography paper (ChrPr, 3001-878, Whatman, GE Healthcare, Chicago, IL, USA) with a hydrophobic area (blue) around the hydrophilic area (sky blue). Device1A and Device1B were fabricated to observe the differences in electrochemical currents due to the electrode arrangements. Device1A has two gold plates as GE and CE on the same side of the ChrPr, as shown in Figure 3a, while Device1B has two gold plates on both sides of ChrPr, as shown in Figure 3b. The Ag/AgCl serves as a reference electrode (RE). Although an auxiliary electrode is used in conventional redox cycling sensor configurations, we omitted it by letting current flow through the reference electrode instead. We will demonstrate that redox cycling occurs only in the structure of Device1B. Meanwhile, Device2 was fabricated to demonstrate a selective measurement of ferrocyanide in the presence of AA by redox cycling as shown in Figure 3c. One major difference between Device1B and Device2 is the shape of hydrophilic area: Device2 has a large circular hydrophilic area beneath the electrodes, minimizing the effects from hydrophilic channel outside the electrode. This structure is effective at minimizing unwanted effects such as the side diffusion of AA into this reaction area.

**Figure 4:**
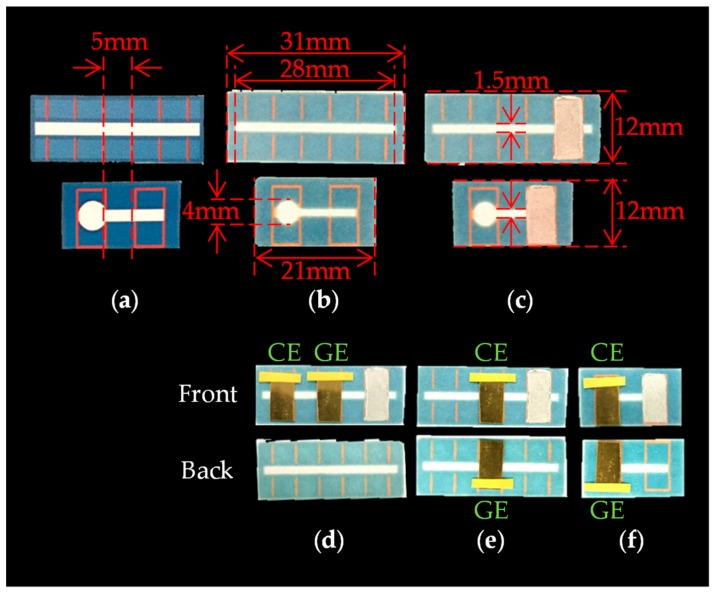
The fabrication process of PBBS: (**a**) printed ChrPr, (**b**) heated ChrPr, (**c**) ChrPr with Ag/AgCl ink, (**d**) Device1A, (**e**) Device1B, and (**f**) Device2.

The fabrication process of PBBS is as follows. The hydrophobic pattern schematized with Microsoft PowerPoint is printed on the ChrPr by a wax printer (Xerox ColorQube 8580) as shown in Figure 4a, and the printed ChrPr is heated in the oven at 120 °C for 60 s. The hydrophobic area is then formed because the wax-ink spreads to the back side of the paper as shown in Figure 4b. As shown in Figure 4c, reference electrode was formed by applying Ag/AgCl ink (011464, BAS, Tokyo, Japan) and air dried overnight. Gold plates (173380, Nilaco, Tokyo, Japan) cut in 5 mm × 10 mm are used as both GE and CE, and they are placed on the ChrPr using vinyl tape as in Figure 4d–f.

Three types of sample solutions were prepared: ferrocyanide, AA, and mixture solutions. The ferrocyanide solution contains a potassium ferrocyanide (K_4_[Fe(CN)_6_]: 165-03745, Wako, Osaka, Japan) in a phosphate-buffered saline (PBS: 10 mM, pH = 7.2~7.4, 164-18541, Wako) and the AA solution contains a l-ascorbic acid sodium (C_6_H_8_O_6_: 196-01252, Wako) in a PBS. Likewise, the mixture solution was prepared by adding the ferro solution to the AA solution.

The 12 μL of the samples were dropped on Device1A and Device1B, and 5 μL was dropped on Device2. Each PBBS was clamped by a clip with a transparent polyvinyl chloride (PVC) plate, which is coated with a stain-resistant PTFE tape (5490R, 3M, Tokyo, Japan). All the electrochemical measurements were performed with Source Measure Unit (SMU: B2902A, Keysight, Santa Rosa, CA, USA) at room temperature by keeping humidity at approximately 80%. 

In CV measurement, the potential of GE was swept between 0 to +700 mV vs. Ag/AgCl at a scan rate of 1.0 mV/s, while the potential of CE was held at a constant reduction potential of −200 mV vs. Ag/AgCl, as shown in Table 1. The scan rate was set low enough to observe the effects of redox cycling under the given electrode spacing, as well as the decreased diffusion coefficient in the paper. Meanwhile, CA measurements were performed by applying a constant oxidation potential (+500 mV) to GE and a reduction potential (−200 mV) to CE.

## 4. Results and Discussion

### 4.1. Characterization of Device1A and Device1B by Cyclic Voltammetry

**Figure 5:**
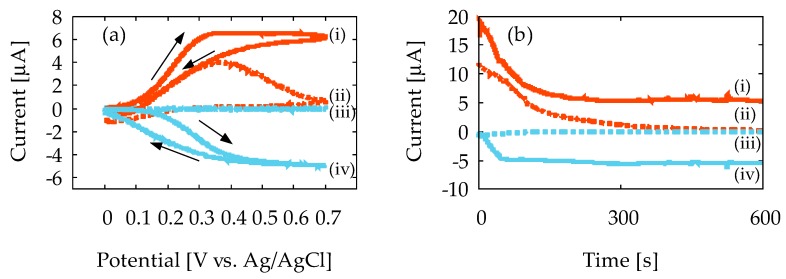
The results of cyclic voltammetry and chronoamperometry measurements with Device1A and Device1B using 10 mM ferrocyanide: (**a**) voltammogram and (**b**) current vs. measurement time. The lowercase roman numbers indicate (i) generator current I_GE_ on Device1B; (ii) generator current I_GE_ on Device1A; (iii) collector current I_CE_ on Device1A; and (iv) collector current I_CE_ on Device1B.

Figure 5a shows the representative result of the CV measurement at 1.0 mV/s sweep rate with the 10 mM ferrocyanide solution, using Device1A and Device1B. In Figure 5a, the vertical axis is the current that flows to the GE and CE, and the horizontal axis is the potential applied at GE. In Device1A, the oxidation current magnitude, I_GE,_ decreases after the current peak is reached, as denoted by (ii) in the figure, and CE does not obtain reduction current, I_CE_, as denoted by (iii). This indicates that the diffusion layer keeps expanding, and ferrocyanide generated at GE does not reach to the CE. Therefore, redox cycling does not occur, and the steady-state current is not observed at all, because the reduction reaction will never occur at CE. In contrast, the CE in Device1B shows a distinct I_CE_, as denoted by (iv), and the magnitude of I_GE_ and I_CE_ at high potential becomes almost constant, as denoted by (i) and (iv). Such sigmoidal-shaped voltammograms are often seen in redox cycling, and imply that the spreading of the diffusion layer has completely stopped in Device1B because of redox cycling. As a result, steady-state current is obtained due to steady-state concentration gradient. Thus, the redox cycling of ferrocyanide with Device1B is confirmed by our experiment. The current amplification in Device1B over Device1A was calculated to be 1.62 from the I_GE_ at the peak in the CV curves. In Device1A, the interelectrode distance is 5.0 mm, and therefore the concentration gradient keeps decreasing due to the spreading of the diffusion layer. In contrast, the interelectrode distance of Device1B is 0.18 mm (180 μm) and the concentration gradient will be fixed in steady-state due to redox cycling at a higher value than Device1A, and therefore a current amplification is observed.

Figure 5b shows the representative results of the CA measurement with 10 mM ferrocyanide solution. In the figure, the horizontal axis is the measurement time. In Device1A, I_GE_ decreases with time and approaches zero, as denoted by (ii). This is a typical result of conventional CA according to Cottrell’s equation in Equation (1). Moreover, I_CE_ is negligibly small, as denoted by (iii). In contrast, Device1B exhibits noticeable I_GE_ and I_CE_, as denoted by (i) and (iv), respectively. Additionally, the steady-state currents are almost the same in magnitude, and collection efficiency of CE is 93%. The effective diffusion coefficient of ferrocyanide in the ChrPr can be estimated from the saturation current in Figure 5b and Equation (3) under the given concentration and electrode size. This turned out to be 1.3 × 10^−6^ cm^2^/s, which is about 1/5 smaller than the widely accepted value in the literature [36]. Such reduction in diffusion coefficient may be interpreted as a mobility reduction due to the presence of the cellulose fiber network. This estimated diffusion coefficient can be used to estimate the time to reach the steady state by Equation (2), and is about 80 s. This is approximately in agreement with what can be observed in Figure 5b.

### 4.2. Quantitative Measurement of Ferrocyanide in Ascorbic Acid by Chronoamperometry with Device2

**Figure 6:**
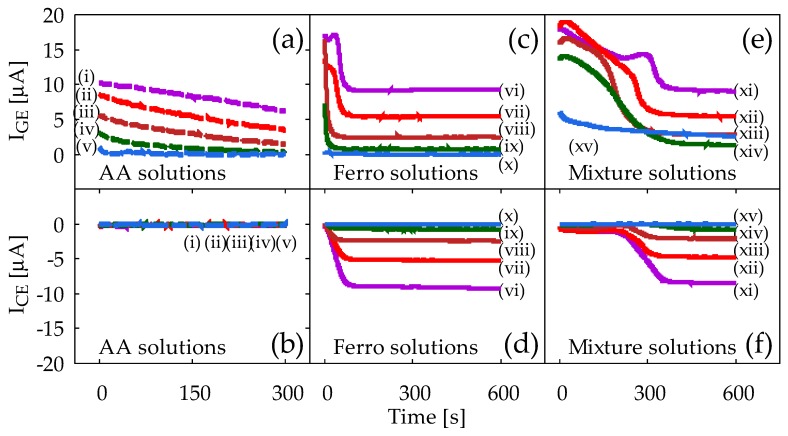
Results of chronoamperometry measurements with Device2 from sample solutions: (**a**,**b**) AA solutions, (**c**,**d**) ferrocyanide solutions, and (**e**,**f**) ferrocyanide/AA mixture solutions. (**a**,**c**,**e**) show currents from the generator electrode, I_GE_, and (**b**,**d**,**f**) show currents from the collector electrode, I_CE_. The lowercase roman numbers in (**a**,**b**) denote AA concentrations (i) 10 mM, (ii) 6 mM, (iii) 3 mM, (iv) 1 mM, and (v) 0 mM. In (**c**,**d**), ferrocyanide concentrations are (vi) 10 mM, (vii) 6 mM, (viii) 3 mM, (ix) 1 mM, and (x) 0 mM. In (**e**,**f**), all mixture solutions contain 10 mM AA, along with ferrocyanide by (xi) 10 mM, (xii) 6 mM, (xiii) 3 mM, (xiv) 1 mM, and (xv) 0 mM. In all measurements, GE and CE are held at +0.5 V and −0.2 V vs. Ag/AgCl reference electrode respectively.

Figure 6 shows results of the CA for ferrocyanide solution and mixture solution measured using Device2. The vertical axis is the current that flows to the GE (I_GE_) and CE (I_CE_), while the horizontal axis is the time after potential application. Measurements were performed five times for each solution to confirm reproducibility, but only representative results are shown.

Figure 6a,b shows the I_GE_ and I_CE_ obtained from the AA solution, respectively. Figure 6a shows a correlation between the concentration of AA in PBS and the obtained I_GE_. Here, the I_GE_ decreases with time, which is a typical result of conventional CA in accordance with Cottrell’s equation in Equation (1). In contrast, Figure 6b shows that no current flows on the CE even with Device2, where the inter-electrode distance is as small as 180 μm. This is, of course, due to the fact that the AA is a chemically irreversible molecule. It is obvious that redox cycling of AA is not seen here at all.

Figure 6c,d shows the I_GE_ and I_CE_ obtained from ferrocyanide solution, respectively. In Figure 6, I_GE_ initially decreases with time, but they finally reach steady-state. Meanwhile, in Figure 6d, I_CE_ start from zero and gradually increase in magnitude until they reach the steady-state current values. The I_CE_ will be observed upon arrival of ferricyanide from GE. Note that the steady-state current values in Figure 6c,d correlates well with the concentration of ferrocyanide, and for each ferrocyanide concentration, the magnitudes of I_GE_ and I_CE_ are almost the same, as can been observed in Figure 5b.

**Figure A1:**
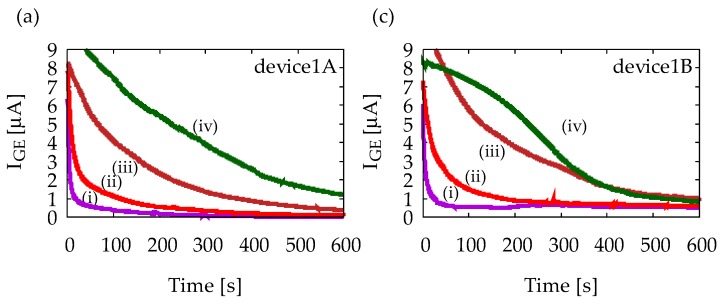

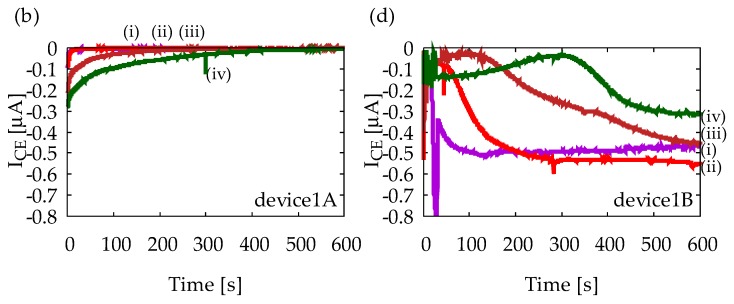
Results of chronoamperometry measurements with (**a**,**b**) Device1A and (**c**,**d**) Device1B. In (**a**,**c**), currents at the generator electrode, I_GE_, are shown, and in (**b**,**d**), currents at the collector electrode, I_CE_, are shown. The lowercase roman numbers indicate different sample solusions: (i) 1 mM ferrocyanide solution, (ii) 1 mM ferrocyanide +1 mM ascorbic acid (AA) mixture solution, (iii) 1 mM ferrocyanide +5 mM AA mixture solution, and (iv) 1 mM ferrocyanide +10 mM AA mixture solution. In all measurements, GE and CE are held at +0.5 V and −0.2 V vs. Ag/AgCl reference electrode, respectively.

Figure 6e,f shows the I_GE_ and I_CE_ obtained from ferrocyanide/AA mixture solution. Compared to the results presented in Figure 6c,d, there are several noticeable similarities and differences. First, it takes a longer time to reach the steady-state than it does in Figure 6c,d: it is around 250–300 s with the mixture solution, while it is around 50–100 s in the ferrocyanide solution. This is attributed to the bulk solution reaction between AA and ferricyanide produced by the electrode reaction. At the beginning of the measurements, the AA in the solution is so abundant that ferricyanide generated at the GE is soon reduced back to ferrocyanide before it diffuses to the CE. This reaction will be effective until all the AA is completely consumed by this reaction itself, along with the oxidation reaction of AA by the GE. Once AA is depleted, regular redox cycling of ferrocyanide starts to appear. The time to reach the steady-state will therefore be dependent on the ratio of the ferrocyanide concentration to the AA concentration in the mixture solution; the smaller the ratio is, the longer the time needed to achieve steady-state current. Such dependence is in fact conceivable in Figure 6e,f, and is also seen in Figure A1c,d for Device1B in Appendix A. Second, the steady-state values of both I_GE_ and I_CE_ are almost the same as those in Figure 6c,d. This implies that the final current values reflect solely the ferrocyanide concentration. This is, of course, due to the fact that all the AA molecules have been converted to DHA, and redox cycling of ferrocyanide alone is playing a major role in steady-state.

The results summarized in Figure 6 thus prove that the redox cycling occurs in our sensor device, and that the concentration of ferrocyanide (reversible) can be selectively measured, even under interference by AA (irreversible). Note that the redox cycling is necessary for such selectivity because otherwise I_CE_ would not have been observed at all, and I_GE_ would have kept decreasing according to Cottrell’s equation in Equation (1), failing to separate I_GE_ due merely to ferrocyanide. The CA results for mixture solution without any redox cycling can be seen in Figure A1a,b for Device1A in Appendix A.

In Figure 6e, the I_GE_ show the abnormal protrusions at around 200–300 s. The larger concentration of ferrocyanide is, the more prominent it becomes. It may be partially because of the resistive voltage drop (IR drop) between the GE/CE and the reference electrode, because it is seen only for the case where the current is larger than 10–15 μA. Although the mechanism of such behavior is not yet clear, there is no noticeable effect on the steady-state current, and therefore it does not compromise our main conclusion.

**Figure 7:**
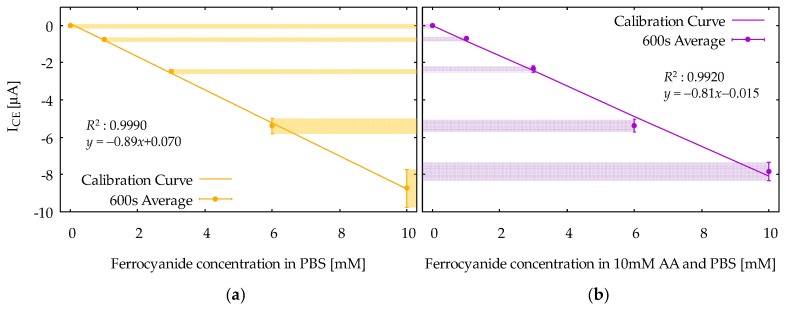
The calibration curves of the reduction current, I_CE_, at *t* = 600 s, as a function of ferrocyanide concentration in (**a**) the ferrocyanide solution and (**b**) the mixture solution. Error bars at each marker represent standard deviations.

Figure 7 shows calibration curves obtained from the I_CE_ at 600 s in CA responses for the ferrocyanide solution (Figure 7a) and the mixture solution (Figure 7b). The vertical axis is the I_CE_, and the horizontal axis is the concentration of ferrocyanide. The calibration curves are based on the average values among the five measurements, and the error bars indicate the standard deviations. Note that the dependence of I_CE_ on ferrocyanide concentration is linear, as is obvious from Equation (3). Moreover, note that the difference between the two calibration curves is relatively small, indicating that the selective measurement of ferrocyanide is successfully realized. From Figure 7b, the limit of detection (LOD) was evaluated to be 16 μM from the formula 3*s*_b_/*m*, where *m* and *s*_b_ are slope of the calibration curve and the standard deviation at zero ferrocyanide concentration, respectively. The calibration curves can also be constructed using I_GE_, but the results would not be different, because the I_GE_ and I_CE_ in the steady-state condition are, in principle, the same in magnitude.

It is interesting to compare the redox cycling performances between Device1B and Device2. In Appendix A, chronoamperometry measurement results with Device1B are shown for the mixture solution containing 1 mM ferrocyanide and 10 mM AA, denoted as (iv) in Figure A1c,d. They can be compared with the results with Device2, denoted as (xiv) in Figure 6e,f. Note that the I_GE_ from Device2 shows a well-defined steady-state current at 600 s, whereas that of Device1B does not yet reach the steady-state completely. In Device1B, AA outside the electrode area can easily diffuse into the electrode area because of the shape of its hydrophilic area, as shown in Figure 3b. This slows down the depletion of AA by the bulk solution reaction described by Equations (4) and (5), and therefore the time to reach the steady-state tends to be long. Such side diffusion of AA is effectively suppressed in Device2, where the hydrophilic area under the electrode has limited access to the side hydrophilic channel, as shown in Figure 3c. 

We used potassium ferrocyanide, which is well-characterized as a target molecule, to demonstrate in principle that our sensor realizes redox cycling. Based on this proven principle, it may be expected to measure dopamine, which is a reversible species. Dopamine is known as a biomarker for Parkinson’s disease, and concentration in human brain or blood is important [38,39]. However, the extremely low concentration of dopamine in blood (sub μM range) under relatively high concentration of AA in blood makes it difficult to measure by electrochemical method [40]. Although such difficulty was overcome by redox cycling [41], sensor fabrication can still be expensive. Apart from further challenges in reducing the paper thickness, our proposed sensor may potentially suggest such high sensitivity redox sensor at extremely low-cost fabrication. Moreover, it may also be used as an enzyme-based glucose biosensor platform by employing enzyme electrodes as one of the generator-collector electrode systems, and using ferrocyanide as an electron mediator [42]. In any case, reduction of measurement time and increase in detection sensitivity will be critical issues to be addressed in future. Both could be overcome by using paper much thinner than 180 μm. The time for molecules to diffuse and reach the other electrode would then decrease dramatically. Moreover, shorter interelectrode distance would cause further enhancement of current response at GE and CE, as predicted from Equation (3). Computer simulation study will give more quantitative prediction of the possibility of our sensor, and it also will play an important role in experimental data analysis and validations.

## 5. Conclusions 

We have demonstrated that the paper-based electrochemical sensor utilizing the redox cycling principle successfully gives electrochemical current dependent only on the concentration of ferrocyanide (reversible redox molecule) even in the presence of interference by the ascorbic acid (irreversible redox molecule). The most notable feature of our sensor is that the redox cycling is realized without any micro/nano fabrication processes, because the paper thickness defines the electrode distance. Selective sensing is possible thanks to the mechanism of (a) redox cycling of reversible redox molecules and (b) depletion of irreversible redox molecules. The working principle of our proposed sensor is expected to be applied to an inexpensive, disposable, and highly-sensitive biomolecule sensor.

## Figures and Tables

**Table 1:** List of measurement parameters.

Measurement Technique	Applied Voltage (mV)		Sweep Rate (mV/s)	Measurement Time (s)
	GE	CE		
CV	0 to +700	−200	1.0	1400
CA	+500	−200		600

## Appendix A

Figure A1 shows chronoamperometry measurement results with Device1A and Device1B using various solutions. All solutions contain 1 mM ferrocyanide, and (i)–(iv) indicates different ascorbic acid (AA) concentrations as (i) 0 mM, (ii) 1 mM, (iii) 5 mM, and (iv) 10 mM. Measurements were performed five times for each solution to confirm reproducibility, but only representative results are shown. Figure A1a indicates that I_GE_ depends on AA concentration, and it decreases in time, which is obvious from Equation (1). Figure A1b indicates that I_CE_ is much smaller than I_GE_ and it does not exhibit any steady-state current. Therefore, Device1A does not show any signs of redox cycling of ferrocyanide. Figure A1c, on the other hand, indicates steady-state currents at 600 s, and their magnitudes are almost the same regardless of AA concentration. Similar steady-state currents are also observed in Figure A1d. Therefore, Device1B exhibits redox cycling of ferrocyanide. However, it is difficult to state that I_GE_ and I_CE_ have completely reached their steady-state. In the structure of Device1B, AA molecules can easily diffuse into the electrode area from the side channels which are outside of the electrode area. The side diffusion of AA from the side channels into the electrode area will slow down the depletion of AA and time to steady-state will become longer. Such side diffusion is suppressed in Device2 by enclosing the area of redox cycling, leading to a better redox cycling performance as shown in Figure 6.
